# Prévalence des staphylocoques à coagulase négative dans les hémocultures au Centre Hospitalier Universitaire Ibn Rochd de Casablanca

**DOI:** 10.11604/pamj.2019.33.193.18552

**Published:** 2019-07-12

**Authors:** Zaineb El Houssaini, Nadia Harrar, Khalid Zerouali, Houria Belabbes, Naima Elmdaghri

**Affiliations:** 1 Laboratoire de Bactériologie-Virologie-Hygiène, Centre Hospitalier Universitaire Ibn Rochd-Casablanca, Casablanca, Maroc; 2 Laboratoire de Microbiologie, Faculté de Médecine et de Pharmacie-Casablanca, Casablanca, Maroc

**Keywords:** Hémoculture, staphylocoque à coagulase négative, contamination, bactériémie, Blood culture, coagulase negative staphylococcus, contamination, bacteremia

## Abstract

**Introduction:**

La réalisation des hémocultures est le meilleur moyen de diagnostic des bactériémies, cependant les résultats faussement positifs peuvent entraîner une confusion concernant les schémas thérapeutiques antibiotiques, mettant ainsi en danger la sécurité des patients. L'objectif principal de ce travail est d'évaluer la prévalence des *Staphylocoques à coagulase négative* (SCN) ainsi que *Corynebacterium spp* et *Bacillus spp* dans les ballons d'hémoculture analysés au laboratoire de microbiologie du Centre Hospitalier Universitaire (CHU) Ibn Rochd de Casablanca. Cette prévalence a été aussi évaluée en fonction de différents services hospitaliers sur l'année 2016.

**Méthodes:**

Il s'agit d'une étude rétrospective descriptive basée sur une analyse de la base de données informatisée du laboratoire de bactériologie-virologie du CHU Ibn Rochd de Casablanca sur une période de 12 mois allant du 1^er^ janvier au 31 décembre 2016, Ont été inclus dans notre étude les bactéries faisant partie de la flore commensale *(staphylocoque à coagulase négative,corynébactéries spp et Bacillus spp)* Les ballons d'hémoculture ont été incubés sur automate Bactec FX. L'identification des germes à partir d'une culture positive a été réalisée selon les techniques standards de bactériologie et l'antibiogramme selon EUCAST 2015. L'étude est basée sur une analyse de la base de données informatisée du système KALISIL (Netika) version (2.2.10.) du Laboratoire de Microbiologie du CHU Ibn Rochd-Casablanca Maroc.

**Résultats:**

Sur 7959 demandes d'hémocultures adressées au laboratoire de bactériologie provenant de 5801 patients, 2491 étaient positifs dont 848, soit 34% des ballons positifs ou 10,6% de l'ensemble des ballons reçus durant l'année 2016, ont été représentées par *staphylocoque à coagulase négative,* 56 soit (2,2%) ballons des hémocultures par *corrynébacteruim SP,* suivi par 60 soit (2,4%) ballons par *bacillus sp.* La fréquence d'isolement du SCN par rapport aux autres bactéries en fonction des services cliniques a montré une fréquence plus élevée dans les services de pédiatrie avec 47,2% suivie des services de médecine avec 44,1%.

**Conclusion:**

Cette étude montre que, Les *staphylocoques à coagulase négative* sont les organismes les plus fréquemment isolés des hémocultures, ils constituent une cause non négligeable d'infections nosocomiales mais, ils sont également les contaminants les plus courants des hémocultures.

## Abstract (en)

**Introduction:**

Blood cultures are the best diagnostic tool for the detection of bacteremia. However, false positive results may lead to confusion about antibiotic regimens, putting the lives of patients at risk. The main purpose of this study was to assess the prevalence of coagulase negative Staphylococci (CoNS) as well as of Corynebacterium spp and Bacillus spp in the bags of blood culture analyzed in the microbiology laboratory at the Ibn-Rochd University Hospital in Casablanca. This prevalence was evaluated according to various Hospital Departments over the year 2016.

**Methods:**

We conducted a descriptive, retrospective study by analysing the computerized database of the Laboratory of bacteriology and virology at the Ibn-Rochd University Hospital in Casablanca over a 12-month period from 1^st^ January to 31^st^ December 2016. Our study focused on bacteria forming part of the commensal flora (coagulase negative Staphylococcus, Corynebacteria spp and Bacillus spp). The blood culture bags were incubated in the automated blood culture system (Bactec FX). The identification of the germs from a positive culture was performed according to the standard techniques of bacteriology and susceptibility testing was performed according to EUCAST 2015. We conducted an analysis of the computerized database of KALISIL system (Netika) version (2.2.10.) of the Microbiology Laboratory at the Ibn-Rochd University Hospital in Casablanca.

**Results:**

Out of 7959 requests for blood cultures obtained from 5801 patients addressed to the laboratory of bacteriology, 2491 were positive, of which 848, reflecting a rate of 34% of positive bags or 10.6% of the whole of bags received over the year 2016, were positive for coagulase negative Staphylococcus, 56 bags of blood cultures, reflecting a rate of 2.2%, were positive for Corrynébacteruim SP, followed by 60 bags of blood cultures, reflecting a rate of 2.4%, which were positive for Bacillus sp. The frequency of isolation of coagulase negative Staphylococcus compared to other bacteria according to Clinical Departments showed a higher frequency in the Paediatric Department (47.2%) followed by the Medicine Department (44.1%).

**Conclusion:**

This study shows that coagulase negative Staphylococci are the organisms most frequently isolated from blood cultures. They are a non-negligible cause of nosocomial infections, but they are also the most common blood culture contaminants.

## Introduction

La pratique des hémocultures est une technique de plus en plus répandue en raison du grand intérêt rapporté dans le diagnostic de la bactériémie d'une part et de la facilité du prélèvement d'autre part cependant, cette voie de prélèvement reste accompagnée d'un risque important de contamination [1, 2] rendant parfois difficile leur interprétation et pouvant conduire à une surestimation de la réalité de l'infection, et donc une hémoculture faussement positive augmente non seulement le travail au laboratoire, mais aussi prolonge le séjour du patient et conduit une utilisation accrue et irrationnelle d'antibiotiques ce qui favorise une pression de sélection des souches résistantes aux antibiotiques et par conséquent, augmente la résistance bactérienne aux antibiotiques. Dans cette étude nous évaluons la prévalence des *Staphylocoques à coagulase négative (SCN)* ainsi que celle du *Corynebacterium spp* et *Bacillus spp* dans les ballons d'hémoculture analysés au laboratoire de microbiologie du CHU Ibn Rochd de Casablanca en se basant sur les résultats du laboratoire puisque nous ne disposons pas de données cliniques des patients hospitalisés. Cette prévalence a été aussi évaluée en fonction de différents services hospitaliers sur l'année 2016.

## Méthodes

C'est une étude rétrospective descriptive, réalisée sur une période allant du 1^er^ janvier 2016 au 31 décembre 2016, qui a colligé l'ensemble des hémocultures provenant de malades hospitalisés dans les Services Cliniques du CHU Ibn Rochd et analysés au laboratoire de bactériologie-virologie du CHU Ibn Rochd de Casablanca. Ont été inclus dans notre étude les bactéries faisant partie de la flore commensale *(staphylocoque à coagulase négative, corynébactéries spp et Bacillus spp).* Les hémocultures sont réalisées lors des pics fébriles à 39-40°C, des frissons, hypotension ou marbrure. Les flacons d'hémoculture aérobie de l'automate du système Bactec Fx (Becton Dickinson) sont inoculés de 10ml de sang veineux pour l'adulte et de 2 à 5ml en pédiatrie, et incubés sous agitation à 37°C. Les flacons négatifs sont rendus stériles après cinq jours d'incubation.

À partir des flacons positifs, nous réalisons un repiquage sur milieu enrichi chocolat et Mackonkey, en parallèle nous faisons un frottis pour la coloration de Gram dont le résultat est communiqué immédiatement au clinicien pour ajuster ou démarrer une antibiothérapie. Après une incubation de milieu ensemencé de 18 à 24H, une coloration de Gram est réalisée à partir des colonies isolées lorsque le Gram est en faveur de Cocci Gram positif en amas, une identification basée sur la catalase et la coagulase pour spécifier les SCN est réalisée, ainsi qu'un antibiogramme en cas de demande de clinicien ou en fonction des renseignements cliniques, à partir de la culture sur des milieux spécifiques. L'étude de la sensibilité aux antibiotiques est réalisée par la technique de diffusion en gélose Mueller-Hinton avec une lecture interprétative selon les normes du comité de l'antibiogramme EUCAST 2015. L'étude est basée sur une analyse de la base de données informatisée du système KALISIL (Netika) version (2.2.10.) du laboratoire de Microbiologie du CHU Ibn Rochd -Casablanca.

## Résultats

**Figure 1:**
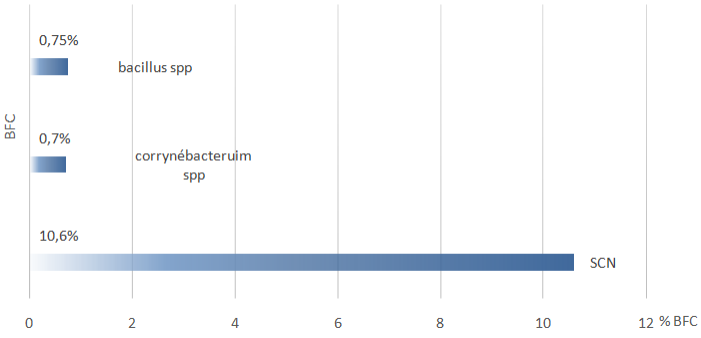
La répartition des bactéries de la flore commensale

**Figure 2:**
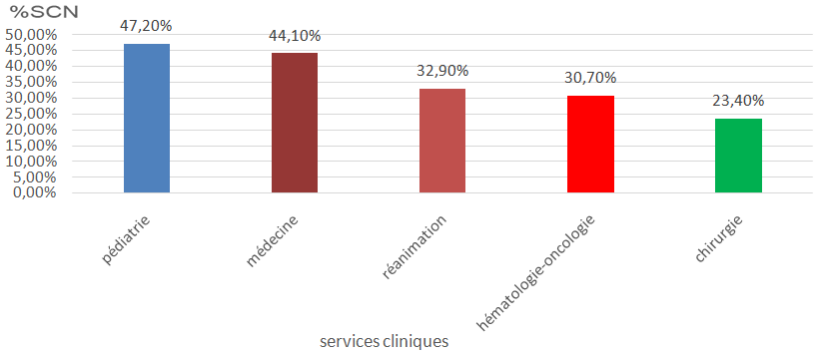
Taux d'isolement de SCN par rapport à l'ensemble des isolats en fonction des services cliniques

Durant la période de l'étude (1er janvier 2016 au 31 décembre 2016), 7959 hémocultures ont été réalisées chez 5801 patients, à raison de 1,3 ballon/patient. Sur la totalité des ballons analysés 2491 étaient positifs soit 27%. Staphylococcus à coagulase négative a dominé le profil des bactéries avec 848 ballons positifs soit 34% des hémocultures positives et 10,6% de l'ensemble des hémocultures réalisées. Les ballons à Corrynébacteruim spp ont représenté 56 ballons positifs soit 2,2% des isolats et 0,7% de l'ensemble des ballons analysés, suivi de Bacillus spp avec 60 ballons positifs soit 2,4% des ballons positifs et 0,75% des demandes réalisées (Tableau 1, Figure 1). La fréquence d'isolement du SCN par rapport aux autres bactéries en fonction des services cliniques a montré une fréquence plus élevée dans les services de pédiatrie avec 47,2% suivi des services de médecine avec 44,1%, les services de réanimation présentent 32,9%, hémato-oncologie 30,7% et enfin les services de chirurgie avec 23,1% (Figure 2).

**Tableau 1:** Nombre et pourcentage d’isolement des bactéries de la flore commensale

Espèce bactérienne contaminant	Nombre de ballons positifs	(%)
SCN	848	34%
Bacillus spp	60	2,4%
Corrynébacteruim spp	56	2,2%

## Discussion

Dans notre étude Les staphylocoques à coagulase négative constituent le groupe de bactéries le plus important parmi les bactéries isolées en hémoculture dans cette série 34% (N=848), cette position d'isolement a été retrouvée dans d'autres études [3, 4]. Le pourcentage d'isolats de staphylocoques à coagulase négative, que nous avons qualifiés de contaminants parmi l'ensemble des ballons reçus et traités en 2016 reste élevé 10,6% par rapport aux données publiées. Le taux de contamination retrouvés dans la littérature est de 2% à 3% [5, 6], mais les taux réels semblent varier considérablement d'un établissement à l'autre [4, 7], selon une étude menée à l'hôpital universitaire de Skane en Suède, de janvier 2006 à décembre 2009, 51 264 ballons hémocultures ont été réalisées chez 14 826 patients. Le taux de contamination était 2,5% de l'ensemble des hémocultures, représentant 38% parmi l'ensemble d'hémocultures positives. Le contaminant dominant était le SCN [8]. Une autre étude a été menée sur l'évaluation prospective des taux de contamination dans 640 laboratoires, un total de 49 7134 ballons d'hémocultures ont été étudiés. Le taux moyen de contamination des hémocultures a été 2,5%, les mêmes auteurs dans une étude ultérieure qui s'est intéressée à 356 laboratoires pour les données trimestrielles entre 1999 et 2003 ont retrouvé un taux de contamination entre 2,15% et 3,67% tout en précisant que le taux de contamination est plus élevé chez les nouveau-nés par apport aux adultes [9]. Par ailleurs, certaines études ont publié des taux de contamination qui peuvent aller de 1% pour certains jusqu'à plus de 5 % pour autres [10].

**Figure 3:**
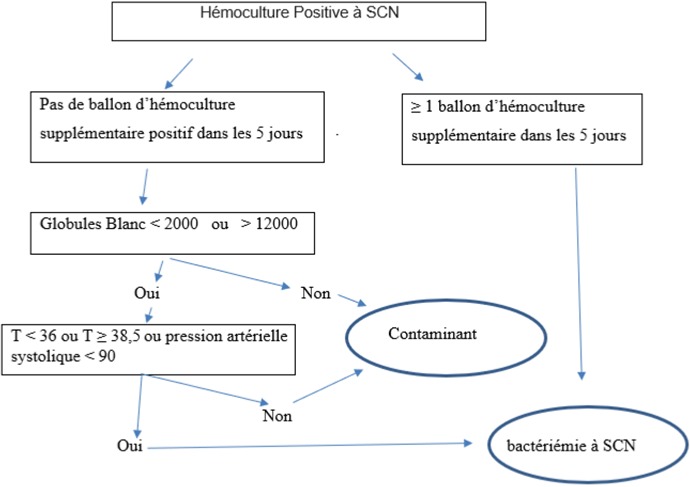
Algorithme optimal pour déterminer la signification des hémocultures positives pour les staphylocoques à coagulase négative (SNC)

Nous devons nous intéresser à ces souches parce qu'il y en a parmi elles celles qui sont responsables d'infections nosocomiales [11-13]. Ce qui rend d'une part l'utilisation des antibiotiques inutiles (en particulier de la vancomycine) et augmente le risque de la résistance bactérienne aux antibiotiques et d'autre part associées à des coûts supplémentaires (pour l'identification du germe et la nécessité de réaliser d'autres examens), l'impact économique se manifeste par l'augmentation des frais et la prolongation de la durée de l'hospitalisations et à un allongement du séjour hospitalier et des traitements antibiotiques inutiles [14-16]. Les hémocultures contaminées provoquent l'incertitude des cliniciens, car il est à première vue, nécessaire d'envisager une réelle bactériémie, en revanche l'échec de la reconnaissance et du traitement de la vraie bactériémie à SCN peut entraîner une augmentation de la morbidité et de la mortalité. Une erreur de trancher entre des épisodes de contamination ou des bactériémies significatives peut également avoir un impact profond sur le taux d'infections sanguines d'un établissement. Néanmoins, différencier les staphylocoques coagulase négatifs pathogènes des contaminants reste difficile car il n'existe pas de moyen sûr pour le faire [17], le taux de 10,6% retrouvé chez nous ne reflète pas en totalité les contaminants à SCN, surement parmi les souches retrouvées il y en a celles qui étaient responsables d'une bactériémie à SCN mais pour lesquelles il y avait des difficultés à l'identifier, Plusieurs auteurs ont proposé diverses définitions cliniques et biologiques pour aider à déterminer la signification clinique de la SCN [13, 16, 18]. Selon une étude faite par Beekmann S.E. *et al.* au Centre Hospitalier Universitaire d'Iowa aux Etats-Unis, un algorithme a été proposé pour déterminer la signification clinique des SCN. Cet algorithme était basé sur l'examen de 405 épisodes de SCN isolés à partir d'hémocultures. Il présente une sensibilité combinée de 62% et spécificité de 91%, et la signification clinique des SCN était défini comme au moins deux hémocultures positives pour les SCN au bout de 5 jours, ou une hémoculture positive plus autres examens cliniques et biologiques, y compris nombre anormal de globules blancs et température ou pression artérielle [15] (Figure 3).

Spécifier le SCN est très important pour confirmer ou éliminer une septicémie liée à cette bactérie qui est classée dans 16 espèces différentes, dans notre étude malheureusement l'identification s'est basée juste sur la catalase et la coagulase, donc il est important de faire une identification complète de l'espèce du SCN, car isoler plusieurs espèces chez le même patient est en faveur d'une contamination, en revanche l'isolement de la même espèce chez le même patient peut confirmer le caractère pathogène du SCN. l'élaboration d'un algorithme facilement utilisable pour déterminer la signification biologique et clinique des staphylocoques à coagulase négative, en se basant sur l'inclusion des données cliniques d'infection avec le nombre d'hémocultures positives pour la même espèce ou des espèces différentes, semble à la fois raisonnable, rationnel et peut être facilement utilisé pour contrôler l'utilisation des glycopeptides (en réduisant l'utilisation inappropriée de la vancomycine pour les hémocultures positives susceptibles de représenter une contamination) ou pour l'évaluation épidémiologique des taux de bactériémie à staphylocoques à coagulase négative. Enfin, il est important de noter que le nombre d'hémocultures positives et le nombre total d'hémocultures effectuées ainsi que les renseignements cliniques sont des outils importants pour déterminer la signification clinique des contaminants cutanés courants [17]. Ainsi, il est recommandé par la littérature d'adopter cet algorithme qui présente une meilleure combinaison de sensibilité et de spécificité [18]. L'implication des SCN au niveau des différents services reste un sujet controversé nécessitant une bonne corrélation clinico-biologique pour faire la part entre une vraie bactériémie et une contamination.

## Conclusion

Ce travail souligne le taux élevé des staphylocoques à coagulase négative des isolats des hémocultures, cela mérite de revoir les bonnes pratiques de réalisation de ces prélèvements et impose impérativement la mise en œuvre d'un algorithme pour déterminer la signification biologique et clinique des SCN pour faire la part entre contaminants et pathogènes.

### État des connaissances actuelles sur le sujet

- SCN sont les germes les plus isolées des hémocultures;
- Ils sont à la fois des contaminants et aussi des agents pathogènes;
- Le taux le plus élevé est trouvé en pédiatrie.

### Contribution de notre étude à la connaissance

- L'application de l'algorithme pour déterminer la signification des hémocultures positives pour les SCN;
- Améliorer les conditions de prélèvement;
- Appliquer les bonnes pratiques de prélèvement à travers la formation continue.

## Conflits d’intérêts

Les auteurs ne déclarent aucun conflit d’intérêts.
